# University Hospital of Clinical and Ophthalmic Surgery, Berlin

**Published:** 1833-04

**Authors:** 


### UNIVERSITY HOSPITAL OF CLINICAL AND OPHTHALMIC SURGERY, BERLIN.

[FROM A CORRESPONDENT.]

### *Aneurism by Anastomosis of the Temporal Artery.*

This form of aneurismal tumor has not been hitherto sufficiently attended to by the greater number of pathologists, although it is not rare in its occurrence. It happens particularly when an artery divides itself into a great number of branches within a short space.

I have observed it most frequently in the branches of the occipital and temporal arteries. In every case, the disease had arisen in very young subjects, which leads one to suppose that they were usually caused by "vitium primæ formationis." In the commencement the disease is manifested by many parts near one another being affected by sensibly-strong pulsations, which, upon close examination, manifestly correspond to the course of the branches of an artery. When the disease progresses further, it changes its original form. There arises, on the more-strongly-pulsating points, knotty swellings, which approach more or less to each other, and, according to the degree of their extension, form variously-sized and irregular tumors, until, from further extensions, it at length forms a cluster-like swelling.

These aneurisms are accompanied with many difficulties and dangers, when they have been allowed to increase uninterruptedly, which especially appertain to the disease in question.

With respect to the curative means, I have since many years experienced that the ligature of the trunk of the affected arteries was frequently insufficient to the attainment of the proposed effect, particularly when this disease affects the branches of the temporal and occipital, owing to the free anastomosis of the frontal, temporal, and occipital, branches with each other, and with those of the opposite side, supplying the aneurism with the quantity of blood which was intercepted by the ligature of the trunks.

Another circumstance also causes the ligature of the principal artery to be unsuccessful, that many of those small tumors have acquired such a degree of self-existence (*selbständiges lebens*), that the adhesive inflammation consequent on the operation is not sufficient to cause the obliteration of those tumors. More certain results from this method are only to be obtained by applying ligatures on not only one trunk, but upon all those which supply the aneurism with nourishment; an operation which would be extremely painful, and might be dangerous, from the violent inflammation of the scalp.

I have, instead of this operation of tying the trunk, adopted another method, in every aneurism of this kind where pressure could be exercised upon an immediate long surface, and always with the best success; which consists in making a deep incision of tolerable length in the middle of the aneurismal swelling, particularly in that part where the tumors pulsate most strongly, are most numerous and projecting, and in that direction which cuts through, in the one incision, the greater number of those tumors; the operator must instantly arrest the violent hemorrhage in the manner hereafter described. Both steps of this operation are much less painful than the tying of the principal artery or arteries, where such careful dissection is necessary, &c. This method preferred by me is much more certain in its results, inasmuch as it is performed upon the affected part, and thereby causes the adhesive inflammation to be much more perfect; further, it renders unnecessary all secondary operations, and, under ordinary circumstances, the cure is effected commonly in the short space of some weeks.

The history of the case which has given rise to the preceding observations, and which was witnessed by Von Walther and other foreign physicians, is as follows:

A boy, aged ten, of a scrofulous habit, suffered a good deal during the last four years (probably from his birth,) from painful throbbings in the right temporal region. On examination, I found there an elastic and very unequal swelling projecting somewhat more than an inch, and towards the circumference rather firmer; the only tumor which pulsated strongly ceased to do so on firm pressure, which caused pain, but whereby the bones could be plainly felt, and the parts reacquired their normal condition; the point of the tumor occupied the middle of the parietal bone, from which point it extended itself on all sides in a circle whose diameter was at least four inches.

From this tumor the head had the appearance as if the right half were larger than the left. When the hair was removed, the patient fixed, and the apparatus for arresting the hemorrhage was ready, I made an incision in the tumor from before backwards, three inches long, by which the greater number and largest of the aneurismal tumors were cut across. A quantity of arterial blood was lost; but immediately a soft sponge, extending beyond the edges of the tumor, was firmly pressed, and the pressure was continued some seconds with undiminished vigour, so that the lips of the wound and contiguous parts were emptied of blood as much as possible. Whilst the operator had one hand engaged by exercising pressure on the sponge, he seized with the other a tolerably large compress, removed the sponge, and at the same moment applied the compress, before hemorrhage could again occur, between the lips of the wound, and then covered the whole tumor with a sponge extending beyond its edges, of about one inch thick, which was made fast by means of adhesive straps and a bandage. After some hours I diminished the pressure which at first was necessary, by undoing some of the folds of the bandage.

On the second day the sponge was replaced by compresses, in order not to cause either too much pain or inflammation by immoderate pressure. Very slight antiphlogistic measures were necessary to subdue some pain and fever consequent on the inflammation.

On the seventh day, the compresses were separated by a copious suppuration, and the wound soon healed, its edges being brought together by adhesive straps. No secondary hemorrhage took place. There was not the slightest trace of pulsation in the circumference of the tumor, and the pain and swelling had entirely disappeared.

